# Rethinking the management of immune checkpoint inhibitor‐related adrenal insufficiency in cancer patients during the COVID‐19 pandemic

**DOI:** 10.1002/edm2.246

**Published:** 2021-03-08

**Authors:** Kevin C. J. Yuen, Michael J. Mortensen, Amir Azadi, Ekokobe Fonkem, James W. Findling

**Affiliations:** ^1^ Departments of Neuroendocrinology and Neurosurgery Barrow Neurological Institute University of Arizona College of Medicine and Creighton School of Medicine Phoenix Arizona USA; ^2^ University of Arizona College of Medicine‐Phoenix Phoenix Arizona USA; ^3^ Departments of Neurology and Neuro‐Oncology Barrow Neurological Institute/Ivy Brain Center at Phoenix St. Joseph’s Hospital and Medical Center Phoenix Arizona USA; ^4^ Division of Endocrinology and Molecular Medicine Medical College of Wisconsin Milwaukee Wisconsin USA

**Keywords:** adrenal insufficiency, cancer, COVID‐19, glucocorticoids, immune checkpoint inhibitors

## Abstract

**Introduction:**

The coronavirus disease 2019 (COVID‐19) is currently a major pandemic challenge, and cancer patients are at a heightened risk of severity and mortality from this infection. In recent years, immune checkpoint inhibitor (ICI) use to treat multiple cancers has increased in oncology, but equally has raised the question of whether ICI therapy and its side‐effects is harmful or beneficial during this pandemic.

**Methods:**

A combination of published literature in PubMed between January 2010 and December 2020, recommended guidelines in non‐cancer patients, and clinical experience was utilized to outline recommendations on glucocorticoid timing and dosing regimens in ICI‐treated patients presenting with AI during this COVID‐19 pandemic.

**Results:**

The potential immune interaction between ICIs and COVID‐19 require major consideration because these agents act at the intersection between effective cancer immunotherapy and increasing patient susceptibility, severity and complications from the SARS‐CoV‐2 sepsis. Furthermore, ICI use can induce autoimmune adrenal insufficiency (AI) that further increases infection susceptibility. Thus, ICI‐treated cancer patients with AI may be at greater risk of COVID‐19 infection. Glucocorticoids are the cornerstone for replacement therapy, and for treatment and mitigation of adrenal crisis and relief of mass effects in ICI‐related hypophysitis. High‐dose glucocorticoids have also been used with cytotoxic chemotherapy as part of cancer treatment, and iatrogenic AI may arise after glucocorticoid discontinuation that increases the risk of adrenal crisis. Furthermore, in patients who develop the “long COVID‐19” syndrome, when to discontinue glucocorticoid therapy becomes crucial to avoid unnecessary prolongation of therapy and the development of iatrogenic hypercortisolemia.

**Conclusion:**

During the COVID‐19 pandemic, much of cancer care have been impacted and an important clinical question is how to optimally manage ICI‐related AI during these unprecedented times. Herein, we suggest practical recommendations on the timing and dosing regimens of glucocorticoids in different clinical scenarios of ICI‐treated cancer patients presenting with AI during this COVID‐19 pandemic.

## Graphical abstract

Cancer patients treated with immune checkpoint inhibitors (ICIs) who develop adrenal insufficiency may be at a greater risk of severity and mortality from the COVID‐19 infection. The effects of ICIs and the appropriate use of glucocorticoids is crucial in optimally managing these patients and preventing unnecessary hypercortisolemia and worsening of symptoms of some patients, including those with long COVID‐19. We suggest practical recommendations on the timing and dosing regimens of glucocorticoids in different clinical scenarios of ICI‐treated cancer patients with AI during this COVID‐19 pandemic.

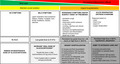

## 1 INTRODUCTION

In December 2019, a novel coronavirus called severe acute respiratory syndrome coronavirus 2 (SARS‐CoV‐2) that originated in Wuhan, China caused an outbreak of coronavirus disease 2019 (COVID‐19) that continues to plague the world and will likely last for a prolonged period.^1^ While often following a mild course, severe cases can present with pneumonia, cytokine release syndrome, and acute respiratory distress syndrome that commonly results in death. Recent studies of COVID‐19 in cancer patients have suggested increased complications and severe outcomes,^2^, ^3^ with older age and treatment with immune checkpoint inhibitors (ICIs) conferring a greater risk.^4^

Immune checkpoint inhibitors (ICIs) have recently revolutionized cancer treatment in the oncology realm^5^ by enhancing the immune response, but may also trigger immune‐related adverse events (irAEs)^6^ that can affect multiple organs, including the skin, colon, lungs, gastrointestinal tract and endocrine glands.^7^ These agents are a unique class of monoclonal antibodies that target specific cell surface proteins involved in immune system inhibition, collectively known as ‘checkpoint inhibitors’.^8^ An explanation underpinning the notion that ICI therapy might exacerbate the course of COVID‐19 infection is linked to the common pathological features between irAEs of ICIs and COVID‐19 that include unrestrained immune^9^ and cytokine hyperactivation.^10^ The growing clinical use of ICIs and the increasing recognition of their irAEs that are radically different from those associated with other cancer treatments means more non‐oncology clinicians will be called upon to manage these patients.^11^ Therefore, rapid and efficient interactions between oncologists, endocrinologists and other medical specialists are required to optimize the management of the diverse irAEs of ICIs.

Glucocorticoids (GCs) are the cornerstone at physiological doses for replacement therapy for AI and at high doses for their anti‐inflammatory effects^12^ for treatment of chronic inflammatory diseases (e.g., asthma, rheumatoid arthritis and inflammatory bowel disease). Glucocorticoids are also used together with ICIs for cancer treatment^13^, ^14^ and by some front‐line clinicians to treat severely ill COVID‐19 patients with diffuse lung damage.^15^, ^16^ The complex interactions of COVID‐19 and cancer patients treated with ICIs who develop ICI‐related primary or secondary adrenal insufficiency (AI) and iatrogenic AI after withdrawal of high‐dose GCs potentially places these patients at a heightened risk of COVID‐19 and adrenal crisis.^17^, ^18^, ^19^ Furthermore, as much of cancer care have been impacted during the COVID‐19 pandemic,^20^ an important question is how to screen, counsel and optimally manage ICI‐related AI during these unprecedented times.

## 2 AIMS AND METHODS

This review discusses the association between COVID‐19 and cancer, debates the appropriateness of ICI use during the COVID‐19 era and provides practical recommendations for clinicians on the timing and dosing regimens of GCs in a variety of clinical scenarios of cancer patients presenting with ICI‐related AI. We utilized a combination of published literature, recommended guidelines in non‐cancer patients, and clinical experience to help outline our proposed recommendations on the timing and dosing regimens of GCs in different clinical scenarios of ICI‐treated cancer patients presenting with AI during this COVID‐19 pandemic.

## 3 COVID‐19 INFECTION, IMMUNE CHECKPOINT INHIBITOR THERAPY AND ADRENAL INSUFFICIENCY IN CANCER PATIENTS

### 3.1 COVID‐19 infection and cancer patients

Cancer patients are more susceptible to infections due to co‐existing chronic diseases, overall poor health status, and systemic immunosuppressive states caused by the cancer and anti‐cancer treatments.^21^, ^22^ The immunosuppressed state of cancer patients (whether caused by the disease itself or the treatment) increases their risk, severity and complications of COVID‐19 infection.^23^ The risk is further exacerbated by the limited access of cancer patients to required health care and inability to receive necessary medical services in a timely manner (especially in high‐risk epidemic areas).^24^ Patients have also been previously advised not to seek medical attention especially during the early phase of the COVID‐19 pandemic because of the increased infection risk.^25^ Therefore, whether to continue or halt anti‐cancer therapy remains debatable, as the risk of cancer progression after stopping cancer therapy remains inconclusive.

### 3.2 Use of immune checkpoint inhibitors and glucocorticoids in the COVID‐19 era

Immune checkpoint inhibitors are monoclonal antibodies that target immune checkpoints (PD1 and PD‐L1), and by virtue of restoring the antitumor immunity through the reversal of immune escape or evasion have led to significant antitumor activity. Because of their mechanism of action in enhancing immune response, specific immune‐related endocrinopathies are increasingly apparent ranging from moderate to severe and life‐threatening ones,^26^ and GCs have been utilized to treat these irAEs.^27^ If the ICI‐treated patient is infected with COVID‐19, there are conflicting opinions about GC use because of their side‐effect profile.^28^ In a recent retrospective study of patients with symptomatic COVID‐19, age above 65 years and treatment with ICIs were predictors for hospitalization and severe disease.^4^ As only one out of 31 patients treated with ICI received GC therapy before the severe illness endpoint, the authors postulated that the ICIs, and not GCs, that was responsible in exacerbating lung injury or triggering immune T‐cell hyperactivation, which in turn induced acute respiratory distress syndrome. When GCs were used to control irAEs in ICI‐treated patients, risk of serious infections increased.^29^ Conversely, in a systematic review, Garant et al.^14^ reported that the type and doses of GCs used with ICI therapy did not lead to poorer outcomes. To date, data on the GC use in ICI‐treated cancer patients remain inconsistent and there are no prospective data to address this concern.

### 3.3 Immune checkpoint inhibitor‐related adrenal insufficiency

#### 3.3.1 Secondary adrenal insufficiency caused by immune checkpoint‐related hypophysitis

Hypophysitis is one of the most common endocrine irAEs associated with ICI therapy.^26^ Adrenocorticotropic hormone (ACTH) deficiency is most frequently reported (20%–75%), followed by LH/FSH (15%–60%), TSH (25%–58%), growth hormone (5%–41%) and prolactin (13%–25%) deficiencies, whereas panhypopituitarism (three or more pituitary hormone deficits) has been observed in up to 50% of cases.^26^, ^30^ Unlike other organs where the side‐effects can often be treated with ICI withdrawal and high‐dose GC therapy, damage to pituitary cells—particularly corticotrophs—are usually permanent.^26^ The incidence of hypophysitis is higher with use of anti‐CTLA‐4 inhibitors such as ipilimumab (0%–17%) than with PD‐1 inhibitors nivolumab and pembrolizumab (0.5%–2.0%),^31^ whereas PD1 and PD‐L1 inhibitors rarely cause hypophysitis^32^, ^33^ but can cause isolated ACTH deficiency.^34^

The clinical manifestations of hypophysitis can range from having none to one or more features of pituitary hormone deficits (e.g., symptoms of hypothyroidism and hypogonadism) to the acute onset of AI (e.g., hypotension, nausea and fatigue) and mass effect symptoms (e.g., headache and visual disturbances that include visual field deficits and ophthalmoplegia).^35^ Additionally, the onset of headache and visual symptoms may be insidious, subacute, or acute and even mimic symptoms of pituitary apoplexy.^36^ The severity of pituitary hormone deficits can be variable as well and may be disproportionate to the MRI findings.^37^, ^38^

Previous studies have demonstrated that non‐cancer patients with AI have a twofold to eightfold higher risk of infection.^39^, ^40^ Although there is currently no published literature of adrenal crisis occurring in patients with ICI‐related hypophysitis, cancer patients are known to be more susceptible to infections,^41^, ^42^, ^43^ a common precipitant of adrenal crisis. Therefore, the risk for developing adrenal crisis is likely to be higher in ICI‐treated patients with AI; hence, it is important that clinicians educate patients about this potential complication and ability to stress dose. Clinicians should be aware that in less severe forms of AI, patients may report non‐specific symptoms of fatigue, malaise or nausea that are common in cancer^44^ and COVID‐19^45^ patients that make differentiating the cause of these symptoms challenging.

#### 3.3.2 Primary adrenal insufficiency caused by immune checkpoint inhibitor therapy

Primary AI due to ICI therapy is uncommon and has been associated primarily with ipilimumab and rarely in combination with other ICIs.^46^ The true incidence of ICI‐related primary AI is difficult to estimate in part because many clinical trials involving ICI therapy report AI without specifying whether the AI is primary or secondary in aetiology. Autopsy studies on patients who died from COVID‐19 infection have shown degeneration and necrosis of the adrenal cortical cells, suggesting a direct cytopathic effect of the SARS‐CoV‐2 virus.^47^ Patients with primary AI may also be at higher risk of adrenal crisis than those with secondary AI due to the lack of mineralocorticoids resulting in a greater risk of dehydration.^48^

#### 3.3.3 Iatrogenic adrenal insufficiency caused by use of exogenous glucocorticoids for other conditions

Cancer patients may develop iatrogenic AI following use of high‐dose GCs as part of chemotherapy or targeted towards suppression of an inflammatory response for other chronic conditions.^49^, ^50^ Excessive GC use is associated with increased risk of infections due to its immunosuppressive actions.^51^ Prolonged use and lifelong requirement using supraphysiological GC replacement doses can cause symptoms of AI after GC discontinuation due to hypothalamic‐pituitary‐adrenal (HPA) axis suppression. A recent meta‐analysis by Broersen et al.^52^ demonstrated that all patients using GC therapy are at increased risk for iatrogenic AI. Differentiating these patients from those with ICI‐induced primary and secondary AI will be important but may be challenging when the high doses of GCs require dose tapering and whether they should be maintained on physiological GC replacement. Therefore, re‐testing and long‐term monitoring of the HPA axis is imperative after completion of GC and ICI therapies, as the axis might recover over time in patients with iatrogenic AI but not likely in those with primary and secondary AI. Because opioids are used frequently in cancer patients,^53^ clinicians should also be mindful of opioid‐induced suppression of the HPA axis, especially those having receiving large opioid doses (morphine milligram equivalent of >20 mg/day) that in turn further increases the risk for adrenal crisis.^54^

## 4 DIAGNOSIS OF ICI‐RELATED ADRENAL INSUFFICIENCY

**FIGURE 1:**
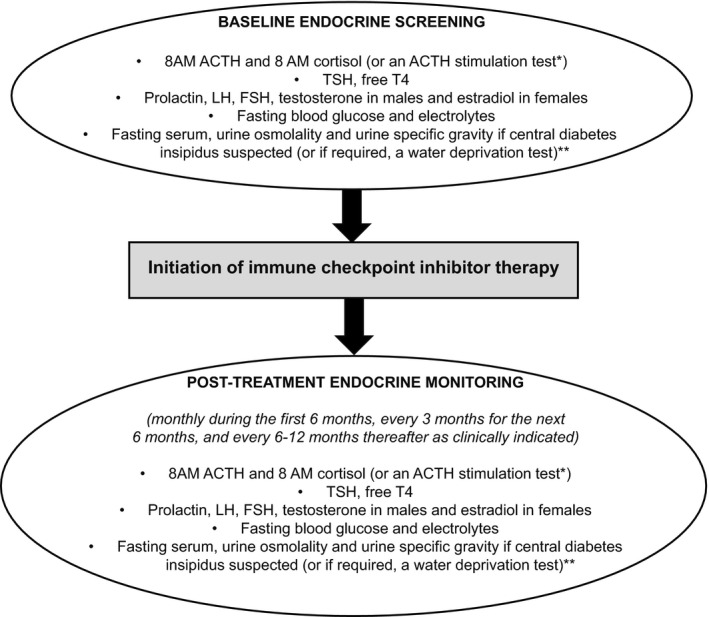
Proposed algorithm for endocrine screening and monitoring in immune checkpoint inhibitor‐treated patients during COVID‐19 pandemic. *Baseline and/or post‐ICI therapy ACTH stimulation test may be considered in cases of equivocal 8 AM serum cortisol levels. Caution must be used in interpreting the results of post‐ICI therapy ACTH stimulation test as it may be falsely normal in the setting of acute ACTH and cortisol deficiencies. **May be performed at the clinician's discretion depending on whether the patient reports symptoms of excessive thirst, polydipsia and hypotonic polyuria

During the COVID‐19 pandemic, it is important that ICI‐related endocrinopathies are not missed. Prior to initiation of ICI therapy, baseline measurements of basal pituitary hormones (8 AM ACTH, 8 AM cortisol, TSH, free T4, FSH, LH, prolactin, testosterone in males or estradiol in premenopausal females), fasting glucose and electrolytes should be obtained (Figure 1).^35^ Depending on whether the patient reports excessive thirst, polydipsia and hypotonic polyuria that raises the possibility of diabetes insipidus, fasting serum, urine osmolality and urine specific gravity may be performed at the clinician's discretion, and if the diagnosis remains equivocal, a water deprivation test may be considered (Figure 1).^35^ Hyponatremia with normokalemia may be present in secondary AI due to excess vasopressin secretion^55^ but hyperkalemia would not be expected as mineralocorticoid secretion is controlled primarily through the renin‐angiotensin‐aldosterone system, which would be intact in these cases.^56^ However, if hyponatremia, hyperkalemia, and elevated plasma ACTH and renin levels in the setting of very low morning serum cortisol levels are found, primary AI is likely.^46^ Conversely, hyponatremia and hypokalemia may occur in cancer patients due to chronic diarrhoea and vomiting from the cancer and/or chemotherapy. Therefore, in the appropriate clinical context and especially in the presence of hyponatremia and hypotension, AM serum cortisol levels <3 µg/dL (80 nmol/L) are highly suggestive whereas levels >15 µg/dL (415 nmol/L) are unlikely to indicate AI.^56^, ^57^, ^58^ Values in between 3 (80 nmol/L) and 15 µg/dL (415 nmol/L) are equivocal and ACTH stimulation testing may be performed if indicated.^56^, ^57^, ^58^ Failure to respond to ACTH stimulation with peak serum cortisol levels of ≤18 µg/dL (500 nmol/L) suggests AI,^56^ although the recent introduction of new, more specific cortisol assays using either monoclonal antibodies or LC‐MS/MS indicate that a lower serum cortisol cut‐point of 14.5 µg/dL (400 nmol/L) may be considered.^59^ After ICI therapy has been initiated, we recommend monthly endocrine monitoring during the first 6 months, every 3 months for the next 6 months and every 6–12 months thereafter (Figure 1).^57^, ^58^ More frequent endocrine monitoring may be required during the COVID‐19 pandemic, especially if symptoms persist or worsen. Basal 8 AM ACTH levels may be helpful to differentiate between primary and secondary AI because treatment for primary AI requires the inclusion of mineralocorticoid replacement in addition to GC replacement, whereas secondary AI requires only GC replacement.

## 5 GLUCOCORTICOID USE FOR CRITICALLY ILL COVID‐19 PATIENTS WITH IMMUNE CHECKPOINT INHIBITOR‐RELATED ADRENAL INSUFFICIENCY

During the COVID‐19 outbreak in the spring of 2020 that severely affected New York City^60^ and the Lombardy region in northern Italy,^22^ high‐dose GC administration was used in the management of patients who developed acute severe respiratory distress syndrome. The rationale for GC use was based on some evidence that the SARS‐CoV‐2 virus induces severe cytokine and chemokine storm, an exaggerated immune response of the host aimed at preventing the invasion of the pathogen, but subsequently cause diffuse lung damage leading to rapid progression of severe respiratory failure.^61^ Hence, such high GC doses are used to utilize its effects in inhibiting immune responses and pathogen clearance, and suppressing lung inflammation.

Glucocorticoids exert both stimulating and inhibitory effects on the immune response according to their timing and circulating levels.^19^ In the early phase of infections, physiological GC levels are required to prime the immune system that activates the HPA axis to increase adrenal cortisol secretion to higher levels to exert immunosuppressive effects to subsequently decrease autoimmunity and cytokine toxicity. The use of GCs for ICI‐related irAEs is generally for two different objectives. The primary and most common use is as an immunosuppressant to counteract the immune system activation intentionally caused by ICI therapy.^57^, ^58^, ^62^

Considering that severe COVID‐19 is associated with increased inflammation and cytokine storm in the latter stages of the infection, treatment with high doses of intermediate/long‐acting GCs (summarized in Table 1) has inevitably raised some concerns. To date, there are no published consensus guidelines on when and how to increase and when to taper GCs to maintenance doses in COVID‐19 patients with ICI‐related AI. Because of the immunosuppressive effects of high‐dose GC therapy, their use was initially discouraged when the COVID‐19 pandemic first emerged.^25^ During the previous SARS epidemic, high‐dose GC therapy resulted in adverse outcomes^63^, ^64^ prompting the World Health Organization to recommend against their routine use in COVID‐19 patients. It is noteworthy, however, that these patients were treated with extremely high GC doses (> 150 mg/day methylprednisolone dose equivalent).^65^ Indeed, the patients were critically ill and/or resistant to conventional treatment and could have contributed to the increased mortality independent of GC exposure. Conversely, several recent studies have demonstrated the contrary, where methylprednisolone,^15^ dexamethasone^16^ and hydrocortisone^66^ improved the outcomes in moderate to severely ill COVID‐19 patients. Possible explanations for the inconsistent data could be due to the heterogeneity of the studies, different aetiologies of the AI and different GC doses and formulations used. High doses of GCs in the early phase of infection can exert negative effects by promoting viral load, but in the second phase of infection, can dampen the cytokine storm by suppressing the hyperactivation of the immune system.^67^ Furthermore, in critically ill COVID‐19 cancer patients without pre‐existing AI, their HPA axis response to the infection may be down‐regulated and unable to compensate to the stress by increasing adrenal cortisol secretion, thus leading to a state of relative AI. The HPA axis plays an important role in stress‐priming the immune response and the lack of compensatory increase in adrenal cortisol secretion in patients with AI predisposes them to adrenal crisis.

**TABLE 1:** Corticosteroid dose equivalents to cortisol

	Equivalent dose^a^ (mg)	Glucocorticoid equivalent (anti‐inflammatory)	Mineralocorticoid equivalent (sodium retaining)
Cortisol	‐	1	1
Short‐acting			
Hydrocortisone	20	1	2
Cortisone acetate	25	0.8	0
Intermediate‐acting			
Prednisone	5	4	0.8
Prednisolone	5	4	0.8
Methylprednisolone	4	5	0.5
Triamcinolone	4	5	0
Long‐acting			
Betamethasone	0.8	25	0
Dexamethasone	0.75	26	0

^a^ Oral or intravenous.

## 6 RECOMMENDATIONS FOR MANAGEMENT OF PATIENTS WITH ICI‐INDUCED ADRENAL INSUFFICIENCY CONTRACTING COVID‐19 INFECTION

The goals of replacement therapy are to provide sufficient GC exposure to support normal physiologic functions, normal volume status, and to avoid excessive dosing causing iatrogenic hypercortisolism and immunosuppression. Because some COVID‐19 symptoms such as fatigue, malaise, nausea and diarrhoea may overlap with common symptoms experienced by cancer patients with AI, this makes it more difficult for patients to know when and how to stress dose. Patients may also not sufficiently stress dose themselves with GCs at the start of the infection, or conversely, stress dose excessively due to fears of adrenal crisis. Therefore, establishing the need and correct timing of stress dose GC administration as soon as symptoms appear, and increasing the doses relative to the degree of inflammatory damage and the desired effect on the immune system is crucial. Patients must be counselled about sick day management on when to start and how to appropriately increase GC dose, and when to taper their doses when the COVID‐19 infection is resolving. Individualizing the stress dose regimen is important, as stress GC dosing is still largely tailored empirically.^18^ Medic Alert card/necklace/bracelet and adequate refills of injectable hydrocortisone or dexamethasone with instructions about its handling should be implemented.^56^ Cancer patients on ICI therapy should be counselled not to hesitate seeking medical assessment if their signs and symptoms worsen. Maintaining good hydration is very important, and consultation and continued follow‐up with an experienced endocrinologist is invaluable.

**FIGURE 2:**
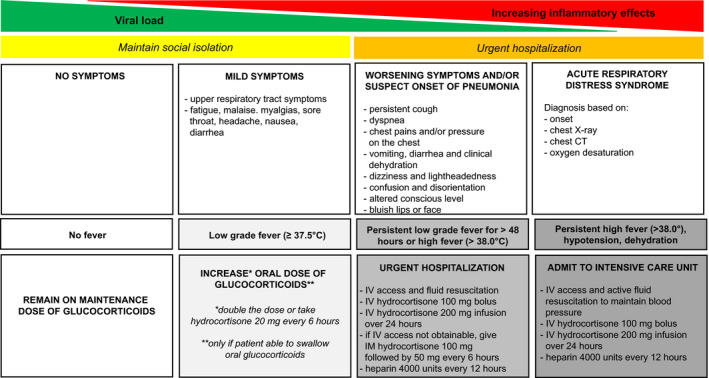
Proposed algorithm for treatment of immune checkpoint inhibitor‐related adrenal insufficiency during COVID‐19 pandemic

Patients who are COVID‐19‐PCR positive and asymptomatic do not require GC dose increments.^68^ We recommend such patients to continue on physiologic GC replacement doses of hydrocortisone (15–25 mg in divided daily doses) or prednisone (5–7.5 mg a day)^68^ without disruption (Figure 2).

In mild‐to‐moderate COVID‐19 infection, we recommend doubling the patient's maintenance GC dose in accordance with ‘sick day rules’,^68^, ^69^, ^70^ and consumption of more electrolyte‐containing fluids as tolerated. (Figure 2) Patients should closely monitor their thirst and urination, and if a low‐grade fever (≥37.5°C) is present, maintenance of adequate self‐hydration, especially for patients with concurrent DI, is recommended. If the low‐grade fever persists >48 h or high fever (>38°C) develops, doubling the GC doses may be insufficient because of relative GC deficiency due to ongoing high levels of inflammation.^69^ In such cases, we recommend to further increase the doses of hydrocortisone but divided to be taken at 20 mg every 6 h to provide more stable GC cover over 24 h.^68^, ^69^, ^70^ (Figure 2) If quarantined at home, patients are advised to obtain sufficient reserves of hydrocortisone and fludrocortisone tablets if availability becomes limited.

If the condition of the patient starts to deteriorate (e.g., worsening dyspnea, chest pains, confusion, disorientation, hypotension or cyanosis), or if the patient cannot eat due to nausea or vomiting, hospitalization is mandatory and close monitoring for features of acute AI (e.g., nausea, vomiting, hypotension and electrolyte imbalances including hyperkalemia, hyponatremia and hypoglycemia) is recommended to avoid adrenal crisis.^68^, ^69^, ^70^ (Figure 2) Such patients require parenteral hydrocortisone treatment of 100 mg followed by 200 mg continuous infusion over 24 h or 50 mg bolus administration every 6‐hourly accompanied by judicious fluid resuscitation.^68^, ^69^, ^70^ This protocol is recommended to cover the amount of GC needed to cope with the inflammation caused by COVID‐19 infection and to reduce the harmful effects that peaks and troughs of GC therapy can inflict to the immune system.^69^ At high doses of GCs, patients with primary AI do not require fludrocortisone as these doses exert adequate mineralocorticoid activity. Hydration and electrolyte balance should be monitored closely and corrected accordingly. Patients with primary AI may be at higher risk of hypovolemia and dehydration than those with secondary AI, especially if their condition has not been adequately treated.^48^ Hence, rehydration with electrolyte replacement (fluids and added salt) is preferable, whereas those with secondary AI, especially with DI, are likely to benefit with more free water. For patients mechanically ventilated, the major stress dose of hydrocortisone of 200 mg/24 h should be continued until mechanical ventilation is discontinued and clinical improvement is observed. When the fever starts to subside and clinical improvement is observed, the GC dose can be tapered back to normal maintenance doses.^68^, ^69^, ^70^

A unique feature of ICI‐treated patients is that they may present with pituitary mass effect symptoms (e.g., headache and visual disturbance) that could also precipitate adrenal crisis. In these circumstances, high‐dose GC therapy (e.g., intravenous or intramuscular bolus injection of 100 mg hydrocortisone followed by continuous intravenous infusion of 200 mg hydrocortisone over 24 h or 50 mg intravenous or intramuscular hydrocortisone injections every 6 h or 1–2 mg/kg/day of prednisone or equivalent) is recommended treat the mass effect^57^, ^58^, ^62^ regardless of whether they have COVID‐19 infection or symptoms of adrenal crisis. If a positive response is observed, perform gradual GC dose taper to replacement doses. In the event of a negative response with or without progressive visual symptoms, surgery should be considered to relieve mass effect and perform GC dose taper to replacement doses.^71^ As soon as there is documented MRI and clinical improvement of the mass effect symptoms, GC doses should then be tapered promptly to avoid undesired GC excess exposure.

It is also important to consider the possibility of patients with AI presenting with adrenal crisis with COVID‐19 being the precipitating factor, and yet display no COVID‐19 symptoms. Thus, in any patient that presents with clinical signs of AI, we recommend performing COVID‐19 testing in these patients, even in the absence of typical COVID‐19 symptoms. Additionally, COVID‐19^72^ and GC^73^ use are risk factors for pro‐thrombotic complications (e.g., microvascular thrombosis, venous thromboembolic disease and stroke), and emergent heparin administration is recommended^74^ as soon as the symptoms escalate from mild to moderate or severe disease (4000 units every 12 h).

Finally, the syndrome of ‘long COVID‐19’ has recently been recognized to refer to patients being ill for more than 4 weeks.^75^ Two groups of long COVID sufferers have been identified: (1) one with mainly respiratory symptoms (e.g., cough and breathlessness), fatigue and headaches; and (2) another group with multi‐organ symptoms (e.g., heart palpitations, gut symptoms, paraesthesia, numbness and brain fog).^76^ Based on data from the RECOVERY trial,^77^ Matthay et al.^78^ surmised that 10‐day dexamethasone therapy of 6 mg daily decreased 28‐day mortality in patients on respiratory support, but patients not requiring oxygen showed no benefit but possibly harmful effects. One rationale for justifying prolonged dexamethasone treatment is the prevention of post‐disease fibrosis in patients susceptible to pulmonary fibrosis. However, prolonged GC therapy can lead to clinical thrombosis^73^ and might contribute to the symptoms of the ‘long COVID‐19’ syndrome. In fact, a meta‐analysis of 21,350 patients with COVID‐19 found that overall mortality was greater among patients treated with GCs (ranging ranged from 3 to 12 days) compared with those not treated with GCs, suggesting that the pro‐thrombotic influence of GCs might have contributed to the increased mortality.^79^ Nevertheless, if dexamethasone is considered in patients with ICI‐induced AI receiving respiratory support, we recommend that treatment can be commenced while being on concurrent hydrocortisone stress doses, but the course of dexamethasone therapy should be no longer than 10 days, as according to the data from the RECOVERY trial,^77^ to minimize the risk of undesired side‐effects caused by prolonged dexamethasone exposure.

## 7 CONCLUSION

This review presents a summary of research, clinical guidance and practical recommendations on the management of ICI‐related AI during the COVID‐19 pandemic. As ICI therapy is a promising therapeutic agent in oncology, its use is increasing but unwanted immune system activation against the endocrine system is an unfortunate reality. In the ongoing COVID‐19 pandemic, one important question is whether cancer patients—already vulnerable to COVID‐19—may be at significantly greater risk of severe COVID‐19 illness if their cancer therapy included ICIs and concurrent AI. The current thinking is that, at least for now, halting or modifying ICI‐related treatment decisions is not justified, and close surveillance with SARS‐CoV‐2 testing is recommended for patients who are on or about to start ICI therapy. Furthermore, knowledge of ICI‐related AI and its potential mass effects due to hypophysitis necessitates GC therapy. However, because symptoms of AI and COVID‐19 are not specific and may be confused with those caused by the cancer, clinicians treating patients with ICI‐related AI should promptly recognize the need to optimize GC therapy in a variety of clinical scenarios. However, GCs is a double‐edged sword in the COVID‐19 setting; hence, they need to be used carefully, considering the risk‐benefit ratio, such as a short treatment course (e.g., not exceeding 10 days) in a select group of COVID‐19 patients for whom survival benefit has been reported. Although some of the symptoms of the ‘long COVID‐19’ syndrome may overlap with those of AI, for example, fatigue and joint pains, there is currently no evidence supporting long‐term GC use to prevent potential adverse sequelae such as pulmonary fibrosis. In this review, we have highlighted several management strategies based on current best practice, but acknowledge the gaps in knowledge of COVID‐19 and the need for more research. It is inevitable that as the prevailing circumstances of the COVID‐19 pandemic continue to change, we will learn more about SARS‐CoV‐2 and that the recommendations for GC therapy will evolve based on developing evidence.

## CONFLICT OF INTEREST

KCJY, MJM, AA, EF and JWF have no conflicts of interest to declare.

## AUTHOR CONTRIBUTION

KCJY conceived and presented the idea for review, and drafted the manuscript. MJM, AA, EF and JWF helped to focus the concept and direction of the review, were involved in reviewing and revising the manuscript critically, and approved the final version of the manuscript.

## ETHICAL APPROVAL

Since this review summarizes and informs already published studies, ethical approval is not applicable.

## Data Availability

There are no new unpublished data associated with this manuscript.
